# Manganese Superoxide Dismutase: Guardian of the Powerhouse

**DOI:** 10.3390/ijms12107114

**Published:** 2011-10-21

**Authors:** Aaron K. Holley, Vasudevan Bakthavatchalu, Joyce M. Velez-Roman, Daret K. St. Clair

**Affiliations:** Graduate Center for Toxicology, University of Kentucky, 454 HSRB, 1095 VA Drive, Lexington, KY 40536, USA; E-Mails: aaron.holley@uky.edu (A.K.H.); vbakt2@uky.edu (V.B.); joycemarievr@hotmail.com (J.M.V.-R.)

**Keywords:** manganese superoxide dismutase, mitochondria, reactive oxygen species, oxidative stress, metabolism, cancer, cardiovascular disease, neurodegenerative disorders

## Abstract

The mitochondrion is vital for many metabolic pathways in the cell, contributing all or important constituent enzymes for diverse functions such as β-oxidation of fatty acids, the urea cycle, the citric acid cycle, and ATP synthesis. The mitochondrion is also a major site of reactive oxygen species (ROS) production in the cell. Aberrant production of mitochondrial ROS can have dramatic effects on cellular function, in part, due to oxidative modification of key metabolic proteins localized in the mitochondrion. The cell is equipped with myriad antioxidant enzyme systems to combat deleterious ROS production in mitochondria, with the mitochondrial antioxidant enzyme manganese superoxide dismutase (MnSOD) acting as the chief ROS scavenging enzyme in the cell. Factors that affect the expression and/or the activity of MnSOD, resulting in diminished antioxidant capacity of the cell, can have extraordinary consequences on the overall health of the cell by altering mitochondrial metabolic function, leading to the development and progression of numerous diseases. A better understanding of the mechanisms by which MnSOD protects cells from the harmful effects of overproduction of ROS, in particular, the effects of ROS on mitochondrial metabolic enzymes, may contribute to the development of novel treatments for various diseases in which ROS are an important component.

## 1. Introduction

Reactive oxygen species (ROS) are by-products of oxygen metabolism [1]. Long thought to have only deleterious effects on cells (damage to lipids, protein, and DNA), ROS have proven to be vital mediators of a host of cellular processes, including cell adhesion, apoptosis, and the immune response [2], as well as cell growth and differentiation [3]. ROS are also important second messengers in intracellular signaling [4,5]. A delicate proportionality exists between ROS production and obliteration, and interruption of this proportionality leads to aberrant ROS accumulation, which contributes to the development of myriad diseases, including various neurological disorders [6] and cancer [7–9].

The mitochondrion is an important metabolic center of the cell. Mitochondria contain many, or all, components of such diverse metabolic pathways as heme synthesis [7], β-oxidation of fatty acids [8], tricarboxylic acid (TCA) cycle [9], amino acid metabolism [10,11], the urea cycle [12], and oxidative phosphorylation to form ATP [13].

Mitochondria, the major oxygen-metabolizing organelles of the cell, are also the major source of ROS in the cell, with the superoxide radical (O_2_^·−^) as the initial ROS produced by this organelle [14,15]. Superoxide radicals can participate in the production of other radicals, including the reactive nitrogen species (RNS) peroxynitrite [16]. ROS can alter cellular function by affecting the activity of myriad proteins, including mitogen-activated [17,18] and serine/threonine kinases [19], protein tyrosine and serine/threonine phosphatases [20], and multiple transcription factors, including AP-1 [21], NF-κB [22], HIF-1 [23], and p53 [24–26]. Metabolic enzymes localized to mitochondria are susceptible to attack by ROS due to their proximity to the sites of ROS production. ROS-mediated changes in the activities of these metabolic enzymes can have remarkable consequences on the homeostasis of the cell. This review focuses on effects of ROS on key mitochondrial enzymes involved in different vital metabolic pathways.

Because ROS are important for normal cellular activities, logic dictates that modulation of basal ROS concentrations, either by increased production of ROS from endogenous sources, exogenous ROS-generating agents, diminished cellular antioxidant capacity, or a combination of the three, will have a dramatic effect on cellular homeostasis and may contribute to disease development. Manganese superoxide dismutase (MnSOD) is the major ROS detoxifying enzyme of cells because of its localization to mitochondria. Altered function or expression of MnSOD can have remarkable consequences on mitochondrial function and the overall health of cells due to oxidative damage to various mitochondria-localized metabolic processes, leading to the development of different diseases [27,28]. This review will focus on important sources of mitochondrial ROS production, the role of MnSOD in scavenging mitochondrial ROS, the effects of ROS on different metabolic enzymes, and the importance of MnSOD in protecting mitochondria from the deleterious effects of mitochondria-derived ROS, with an emphasis on disease development.

## 2. Mitochondrial Production of ROS

### 2.1. Mitochondria is a Major Source of ROS

Mitochondria are the main source of ROS (particularly superoxide radicals) in the cell due, in part, to the oxygen metabolism that occurs at this organelle [29,30]. Multiple enzymes in the electron transport chain are responsible for superoxide production [31], with complexes I (NADH-ubiquinone oxidoreductase) [32,33] and III (ubiquinol-cytochrome *c* oxidoreductase) [34] as major sites of superoxide production. For complex I, the site of superoxide generation was pinpointed to the region between the ferricyanide and ubiquinone reduction sites [35] and was further refined to the iron-sulfur centers N1a [36] and N2 [37]. The proton-pumping activity of complex I is another vital source of superoxide production. The proton pump inhibitor EIPA increases superoxide production at complex I considerably and enhances rotenone-mediated superoxide production [38]. Superoxide production at complex III involves the ubisemiquinone intermediate of the Q-cycle [34], and superoxide is released on both sides of the inner mitochondrial membrane [39] into both the matrix [40] and the intermembrane space [41].

Complex II also adds to the total amount of superoxide radicals produced by mitochondria. The site of superoxide production at complex II was suggested to be distal to the site of succinate oxidation [42], and was identified as either the reduced cytochrome *b*_566_ or ubisemiquinone of the of the Q_0_ site of the cytochrome *bc*_1_ complex of Complex II [43].

Other enzymes within the mitochondria not directly tied to the electron transport chain are also sources of mitochondrial ROS. α-ketoglutarate dehydrogenase (α-KGDH), an important component of the citric acid cycle, contributes to ROS formation in a way that is dependent on the NADH/NAD^+^ ratio [44], with the dihydrolipoyl dehydrogenase component of α-KGDH as the major site of ROS production [45]. Dihydroorotic dehydrogenase (a component of pyrimidine synthesis) produces superoxide as a byproduct of oxidizing dihydroorotate to orotate [46,47]. Glycerophosphate dehydrogenase [48,49] and cytochrome P450s [50,51] also contribute to total ROS production by mitochondria.

Superoxide radicals contribute to the production of other reactive oxygen species that further damage mitochondria. Mitochondria possess many proteins with iron-sulfur centers that are susceptible to attack by superoxide, resulting in the release of free iron cations into the mitochondria. Iron cations participate in the production of hydroxyl radicals from hydrogen peroxide through the Haber-Weiss reaction [52–55]. Superoxide radicals also react with mitochondrial nitric oxide to produce peroxynitrite, a reactive nitrogen species (RNS) [56]. Peroxynitrite can modify various amino acids in proteins, including oxidation of sulfhydryl groups on proteins [57] and the nitration of tyrosine residues [58]. Mitochondrial enzymes are no exception to attack by peroxynitrite [59], with such diverse targets as electron transport chain components complex I [60–63], complex II [61,63], and complex V [61], as well as glutathione peroxidase [64], aconitase [65,66], and MnSOD.

### 2.2. Ways to Scavenge Mitochondrial ROS

Because of the deleterious effects of ROS, the cell is equipped with several enzyme systems to detoxify ROS produced throughout the cell [67,68]. Superoxide dismutases are the major ROS detoxifying enzymes of the cell [69] and catalyze the dismutation of superoxide radicals to hydrogen peroxide and molecular oxygen [70]. Glutathione peroxidase [71,72], peroxiredoxins [73], and catalase [74] decompose hydrogen peroxide generated by SODs to water. Three types of SOD are expressed by cells, encoded by separate genes (reviewed in [75]). Copper- and zinc-containing SOD (CuZnSOD, SOD1) is a homodimer primarily localized to the cytoplasm [76], though, small amounts of CuZnSOD have been identified in the intermembrane space of mitochondria [77,78]. Extracellular SOD (ECSOD, SOD3) shares significant amino acid homology with CuZnSOD (40–60%), contains both copper and zinc in its active site, but is localized to the extracellular region of the cell [79,80]. MnSOD is a homotetramer [81–83] localized exclusively in the mitochondrial matrix [77,78] and is found in multiple organisms, including *Saccharomyces cerevisiae* [82], the red alga *Porphyridium cruentum* [84], *Escherichia coli* B [85], and chicken liver mitochondria [78].

Hydrogen peroxide, while not a radical, is also a ROS, and the cell has developed many enzyme systems to catalyze the decomposition of hydrogen peroxide to water and molecular oxygen [67]. Two forms of peroxiredoxin (PRX) exist in mitochondria [73]: PRX III [86,87] and PRX V [88]. Thioredoxin is used by peroxiredoxins to decompose hydrogen peroxide, generating water and oxidized thioredoxin in the process. Reduced thioredoxin is regenerated by thioredoxin reductase II [89,90]. Glutathione peroxidase (GPX) is another enzyme that removes hydrogen peroxide from the cell. GPX1 [71] and phospholipid-hydroperoxide GPX [72] (PHGPX) are the two mitochondrial forms of GPX, with GPX1 localized to the matrix and PHGPX embedded in the inner membrane [91]. GPX uses glutathione (GSH) to reduce hydrogen peroxide to water, generating oxidized glutathione (GSSG) in the process. GSH is regenerated from GSSG by the enzyme glutathione reductase [92]. Catalase also scavenges hydrogen peroxide in the cell [93,94], however, controversy exists concerning the localization of catalase in the cell. Some labs report catalase in the nucleus, peroxisomes, and the sarcoplasm, but not mitochondria, in mice overexpressing catalase [95]. Other labs have discovered catalase not only in mitochondria [96], but in the matrix of mitochondria [97].

## 3. MnSOD is Essential for Aerobic Life

Numerous studies in different model systems demonstrate the indispensable role for MnSOD in protecting aerobic life from the deleterious effects of oxygen. Gregory and Fridovich [98] discovered that *E. coli* B cells grown under 100% oxygen were more resistant to hyperbaric concentrations of oxygen (20 atm) than *Bacillus subtilis* or cells grown under normal atmospheric conditions due to oxygen-stimulated expression of MnSOD. These *E. coli* B cells were also more resilient against exposure to the superoxide-generating antibiotic streptonigrin. Similar results were seen with the yeast strain *Saccharomyces cerevisiae* var. *ellipsoideus* [99].

Complete knockout of MnSOD has no effect on embryonic development, but leads to death shortly after birth. Depletion of MnSOD enzymatic activity by expression of inactive mutants or knockout of MnSOD gene expression causes early death in mouse [100] and *Drosophila* models [101] due, in part, to decreased activity of various mitochondrial proteins [100,102] (see Effects of MnSOD on Mitochondrial Integrity/Function below). In a study using heterozygous MnSOD knock-out mice (with a 50% reduction in MnSOD enzyme activity in all tissues) [103], there is an increase in oxidative DNA damage (as measured by formation of 8-oxodeoxyguanidine) in nuclear and mitochondrial DNA compared to wild-type mice. This increase in oxidative DNA damage was age-dependent, however, other markers of aging (cataract formation, immune response, *etc*.) were not affected. Interestingly, a 100% increase in cancer incidence was observed in MnSOD heterozygous knockout mice compared to wild-type controls. In a study by Copin *et al.* [104], mice were generated that simultaneously overexpressed CuZnSOD and were deficient in MnSOD. Overexpression of CuZnSOD did not compensate for the neonatal lethality caused by decreased MnSOD expression, suggesting that localization of the antioxidant enzymes is vital to oxidative-stress related cellular damage. In a recent study in *Drosophila*, Mukherjee *et al*. found that while MnSOD is essential for the viability of adult flies, the lack of MnSOD has no effect on embryogenesis or later development and differentiation [105], which seems to be consistent with mouse models, in which homozygous MnSOD knockout mice are of normal size and have no detectable gross deformities at birth [100]. The increased death rate observed in MnSOD homozygous knockout neonates compared to heterozygous knockouts and wildtype littermates may be due to the inability to these animals to compensate for exposure to higher oxygen levels in the atmosphere compared to oxygen levels experienced in the uterus. Various anatomical abnormalities contribute to early death observed in MnSOD knockout mice. Li *et al*. generated a strain of mice expressing an enzymatically inactive mutant of MnSOD and identified several cardiovascular abnormalities in homozygous MnSOD knock-out animals, including dilated left ventricular cavity, reduced left ventricular wall thickness, and myocardial hypertrophy resulting in dilated cardiomyopathy [100]. Lebovitz *et al*. [106] generated a line of MnSOD knockout mice (SOD2^m1BCM^/SOD2^m1BCM^) by deleting exons 1 and 2 from the MnSOD gene. These mice live up to three weeks after birth, but suffer from severe anemia due to lower levels of all hematopoietic cells, resulting in hypocellular bone marrow. Cardiac injury is also observed in the SOD2^m1BCM^/SOD2^m1BCM^ mice, with approximately 10% of the animals demonstrating cardiac injury due to balloon-like cardiac dilation and ventricular wall thinning.

Conditional knockout of MnSOD using the Cre-Lox system has proven valuable in determining the tissue-specific effects of reduced MnSOD. Ikegami *et al*. developed a MnSOD flox mouse, and used this mouse to develop a liver-specific MnSOD knockout model. The researchers found that knockout of MnSOD in the liver had no effect on morphology and there was no increase in oxidative damage as determined by lipid peroxidation, suggesting that either the liver contains compensatory mechanisms to protect from oxidative stress or the liver is susceptible to systemic oxidative stress [107]. Kidney-specific knockout of MnSOD has no effect on lifespan, but does result in a decrease in body weight compared to Cre control mice. Interestingly, while MnSOD knockout mice exhibit altered kidney morphology and increased tyrosine nitration of kidney proteins, there was no overall renal dysfunction compared to Cre control mice [108]. Specific knockdown of MnSOD in type IIB skeletal muscle in C57/BL6 mice results in a significant elevation in mitochondrial superoxide production, causing an increase in oxidative damage. This damage leads to a decrease in the ability of the gastrocnemius and extensor digitorum longus muscles to produce force over time, as well as decreases the aerobic exercise capacity in these mice compared to controls [109]. Interestingly, MnSOD knockdown in type IIB skeletal muscle does not affect age-dependent muscle atrophy [110]. Conditional knockout of MnSOD in postnatal neurons does not result in an increase in oxidative damage, even one year after birth, but does lead to an increase in disorganization of distal nerve axons after injury [111].

Logic suggests that if a decrease in MnSOD has deleterious effects, then overexpression of MnSOD may be beneficial. In a study by Jang *et al.* [112], the effects of MnSOD overexpression on age-related biomarkers was investigated. While overexpression of MnSOD causes an increase in aconitase activity, a decrease in age-related decline in mitochondrial ATP production, a decrease in lipid peroxidation, and protected the mice from paraquat-induced oxidative stress, there were no statistically significant differences between wild-type and MnSOD-overexpressing mice with respect to lifespan or age-related pathology. Likewise, overexpression of the antioxidant enzymes CuZnSOD, catalase, or a combination of CuZnSOD with either catalase or MnSOD does not increase the life span of mice [113]. On the other hand, induction of MnSOD expression in *Drosophila melanogaster* caused an increase in the mean life span of the animals without affecting overall oxygen consumption [114].

## 4. Effects of MnSOD on Mitochondrial Integrity/Function

### 4.1. Electron Transport Chain

Complexes I, II, and III are sources of superoxide (see Mitochondria are a Major Source of ROS, above) and potential victims of the very superoxide they produce, in part, due to the presence of iron-sulfur centers in key subunits of all three complexes [115–120]. These complexes are also susceptible to oxidative modification of key amino acids by superoxide and other ROS/RNS, which can affect their activities. For example, in a study by Chen *et al*. on the complex I subunit NADH dehydrogenase, Cys206 and Tyr177 were two amino acids susceptible to oxidative modification, causing a decrease in the electron transport function of complex I [121], and this type of auto-oxidation of complex I may be involved in Parkinson’s disease [122]. Complex I [123–126] and complex III [126] are also vulnerable to inactivation by peroxynitrite, the product of the reaction of superoxide and nitric oxide. Inactivation of electron transport complexes by ROS/RNS may be involved in such diverse pathological conditions as cellular damage associated with ionizing radiation [126] or the development and progression of various neurological disorders [122,123,125].

MnSOD is important for the scavenging of superoxide generated by the electron transport chain complexes and may be important in preventing ROS-induced inactivation of these complexes. Knockdown of MnSOD in myriad model systems demonstrate altered activity of complexes I, II, and III. Complete knockout of MnSOD in mice causes a substantial reduction in succinate dehydrogenase (complex II) levels in heart tissue [100]. Williams *et al*. show that mitochondria isolated from heterozygous MnSOD knockout mice (MnSOD^−/+^) have a decrease in the respiratory control ratio (RCR) compared to wildtype mice (MnSOD^+/+^). The decrease in RCR was greatest for the complex I substrate glutamate/malate and the complex III substrate duroquinol, and the diminished RCR was linked to a decrease in state III respiration. The authors found a decrease in complex I activity in MnSOD^−/+^ mice compared to MnSOD^+/+^, and this decreased activity was due to oxidation of the iron-sulfur center of complex I [127]. Using wildtype and MnSOD^−/−^ mouse erythroblasts, Martin *et al*. identified by microarray several nuclear gene-encoded subunits of all five complexes of oxidative phosphorylation that were all downregulated in MnSOD^−/−^ erythroblasts compared to wildtype cells [128]. Larosche *et al*. used mice that were either heterozygous MnSOD knockout (MnSOD^+/−^), wildtype for MnSOD, or overexpressing mouse MnSOD (MnSOD^+++^) to better understand the role of MnSOD in the effects of acute ethanol exposure. The researchers found that in wildtype and MnSOD^+/−^ mice, there was a significant decrease in the activities of both complex I and complex V, while these effects of ethanol were completely blocked in MnSOD^+++^ mice. Ethanol administration also resulted in an increase in iNOS expression in all three genotypes, but nitration of complexes I and V was only observed in MnSOD^+/−^ mice. These results demonstrate an important role for MnSOD in modulating peroxynitrite formation, and subsequent nitration of susceptible proteins, in part, by removing excess superoxide that may contribute to peroxynitrite formation [129].

This laboratory has conducted much work concerning the off-target effects of the cancer chemotherapeutic drug adriamycin, especially cardiac and neurological toxicities. A major side effect of anthracycline chemotherapy in cancer patients, especially adriamycin, is a dose-dependent cardiotoxicity [130] that eventually leads to dilated cardiomyopathy and congestive heart failure [131]. The mitochondrion is an important target of adriamycin [132], and because excessive ROS production is a mechanism of adriamycin-induced cardiotoxicity, alterations in the capacity of cells to scavenge ROS can impact the cardiac injury caused by adriamycin [133–135]. Using transgenic mice overexpressing various amounts of MnSOD, this laboratory was the first to demonstrate that mitochondrial ROS is vital for adriamycin-induced cardiac injury [136]. In non-transgenic animals, adriamycin causes a significant reduction in the respiratory control ratio and state III respiration at complexes I and II. In mice overexpressing MnSOD, only complex II activity is affected, implying that MnSOD protects complex I from deactivation caused by adriamycin-induced superoxide production [137].

Another potential side effect of cancer chemotherapy is cognitive decline characterized by memory loss, decreased reaction time, and diminished concentration [138,139], sometimes referred to by patients as chemobrain [140,141]. Adriamycin can cause changes to both the structure [142,143] and activity [144] of various regions of the brain, and oxidative stress is thought to be an important mechanism of cognitive dysfunction associated with chemotherapy [145,146]. This laboratory was the first to demonstrate a unique mechanism of adriamycin-induced neurotoxicity. Adriamycin does not cross the blood brain barrier [147], but it does cause a substantial increase in levels of TNF-α in serum, whole brain homogenate, and increased TNF-α staining in the hippocampus and cortex of mouse brain. Like the effects observed in cardiac tissue, adriamycin causes a significant reduction in state III respiration due to decreased complex I activity [148]. Another important mechanism of adriamycin-induced neurotoxicity is modulation of MnSOD enzyme activity. Adriamycin treatment leads to increased nitration of MnSOD and a corresponding decrease in MnSOD enzyme activity. The effects of adriamycin on MnSOD were not observed in iNOS knock-out mice, implying both a role for iNOS in adriamycin-induced neurotoxicity and the importance of MnSOD in preventing this type of chemotherapy-mediated injury [145].

### 4.2. Tricarboxylic Acid (TCA) Cycle

The TCA cycle (also known as the Krebs cycle) is a vital metabolic pathway in mitochondria, providing reducing equivalents that are fed into the electron transport chain for ATP production and generating substrates used in a variety of cellular processes. Altered activity of various enzymes in the TCA cycle has been linked to different neurological diseases and cancer [9,149].

Aconitase, the enzyme that catalyzes the conversion of aconitate to isocitrate [9], contains iron-sulfur centers and is susceptible to deactivation by superoxide [65,150,151] leading to release of Fe(II) from the enzyme (reviewed in [152]). Aconitase is also sensitive to inactivation by other ROS/RNS, such as *S*-nitrosoglutathione [153] and peroxynitrite [66,153,154]. Peroxynitrite inactivates aconitase by attacking two sites within the enzyme. Low levels of peroxynitrite inhibit aconitase activity, in the absence of citrate, by attacking the iron-sulfur center, converting the [4Fe-4S] center to a [3Fe-4S], with a loss of iron. Much higher concentrations of peroxynitrite are required for inactivation when aconitase is in the presence of citrate. The mechanism of inactivation in this case is oxidation of cysteine 385, which binds to the iron-sulfur center, to sulfonic acid, as well as nitration of key tyrosines and oxidation of cysteine residues to sulfonic acid near the active site of the enzyme [154].

Several studies in various models demonstrate the importance of MnSOD in maintaining aconitase activity, including *Arabidopsis thaliana* [155], yeast [156], *Drosophila* [101], and mouse [127]. For example, microarray analysis of normal and MnSOD^−/−^ mouse erythroids found that complete knockout of MnSOD leads to a significant decrease in the expression of nearly all enzymes and regulatory proteins involved in the TCA cycle [128]. In liver mitochondria from MnSOD^+/−^ mice, aconitase activity was significantly reduced and was rescued by addition of iron and dithiothreitol, indicating that inactivation of aconitase may be due to oxidation of the protein by superoxide radicals [127]. In A549 human lung adenocarcinoma cells, overexpression of MnSOD prevented inactivation of aconitase induced by hypoxia-reoxygenation of the cells [157], as well as the redox-cycling agent phenazine methosulfate and electron transport chain inhibitors [150].

### 4.3. Iron Metabolism

The mitochondrion is vital for proper iron handling and utilization [158] and is the site of two important iron-consuming processes: the synthesis of iron-sulfur centers [159] and heme [7]. Mitochondria are also important for intracellular iron storage by virtue of the presence of a mitochondria-specific ferritin (reviewed in [160]). Improper sequestration and use of iron can lead to iron-induced oxidative damage [161] and is associated with several disorders, including X-linked sideroblastic anemia, Friedreich ataxia, hereditary myopathy, and X-linked sideroblastic anemia with ataxia [162,163].

Altered expression or mutations of MnSOD can have dramatic effects on cellular iron handling. Using electron paramagnetic resonance (EPR), Srinivassan *et al*. demonstrated that knockdown of CuZnSOD, MnSOD, or both in yeast results in a dramatic increase in the amount of EPR-detectable iron compared to wild-type yeast [164]. The Ala16Val polymorphism of the *Sod2* gene (rs4880) is a common single nucleotide polymorphism, and the Ala-MnSOD variant is associated with an increase in intracellular iron in patients with alcohol-induced liver cirrhosis and an increased risk of hepatocellular carcinoma. Transfection of the Ala-MnSOD into the Huh7 human hepatoma cell line increases the expression of cytosolic ferritin, transferrin receptors-1 and -2, hepcidin, and frataxin, proteins involved in different aspects of iron handling [165]. Complete knockout of MnSOD in mouse erythroblasts causes a significant increase in transferrin mRNA [128].

The ATP binding cassette subfamily B member-7 (ABCb7) is another iron-processing protein affected by MnSOD. ABCb7 is a transporter protein localized to the inner mitochondrial membrane [166], is important for export of iron-sulfur centers from mitochondria to the cytosol, and is essential for the formation of cytosolic iron-sulfur center-containing proteins [167,168]. Mutations or loss of ABCb7 are linked to sideroblastic anemia with ataxia [168–170] and mitochondrial accumulation of iron [170–172]. Knockout of MnSOD in erythroid cells is associated with a decrease in the expression of ABCb7 [128]. These studies suggest that decreased MnSOD expression or activity causes accumulation of iron in cells, specifically within mitochondria. Increased MnSOD expression or activity may prove valuable for the treatment of iron toxicity caused by numerous diseases.

Changes in iron metabolism can affect MnSOD expression. Work by Pinkham *et al*. identified *SOD2* as a heme-responsive gene, and heme-dependent regulation occurs through the presence of three cis elements involved that are bound by Hap1p, a heme-binding transcription factor [173]. Heme oxygenase-1 (HO-1) is an enzyme involved in the rate-limiting step of heme degradation: the cleavage of the meso carbon bridge of heme to form biliverdin-IX, carbon monoxide, and free iron. HO-1 is found in mitochondria, where it regulates mitochondrial heme content and the expression of several genes, including cytochrome *c* oxidase subunit I and mitochondrial nitric oxide synthase [174]. Transfection of cultured rat astroglial cells with HO-1 induces MnSOD expression, which was attenuated by treatment with various antioxidants, suggesting a role for oxidative stress in MnSOD expression stimulated by HO-1 [175]. Jiralerspong *et al*. found that in primary fibroblasts, iron-induced expression of MnSOD is impaired in fibroblasts isolated from Friedreich ataxia patients compared to fibroblasts isolated from normal patients. Only high levels of iron could induce MnSOD expression in the Friedreich ataxia fibroblasts, and this induction occurs through an NF-κB-independent mechanism. [176] These studies imply that iron metabolism and MnSOD expression are tightly regulated, that a feedback mechanism is present in cells to carefully balance iron metabolism and oxidative stress, and diseases can result from an upset of this balance.

### 4.4. Apoptosis

Apoptosis is a tightly regulated type of cell death that targets single cells or small groups of cells. Cells first undergo condensation of the cytoplasm and nucleus, leading to the formation of apoptotic bodies, small membrane-bound fragments containing cellular components. Healthy cells surrounding the apoptotic cell then phagocytose the apoptotic bodies [177,178]. Apoptosis is important for a multitude of cellular processes, including the immune response and embryonic development [179]. Apoptosis occurs by two different pathways: extrinsic and intrinsic. During extrinsic apoptosis, the cell receives an external stimulus for cell death [180], while some internal stress triggers the intrinsic pathway of apoptosis [181,182].

Mitochondria are important sites for the initiation and progression of apoptosis [183,184]. Changes in mitochondrial membrane potential, permeabilization of the mitochondrial membrane, and ROS generation can all trigger apoptosis [185]. Upon mitochondrial dysfunction, many molecules are released from mitochondria to initiate and propagate apoptosis. Cytochrome *c*, when released into the cytoplasm, interacts with Apaf-1 to form a protein complex called the apoptosome, which promotes activation of caspase 9. Omi/Htr2 and Smac/Diablo interfere with various members of the inhibitor of apoptosis (IAP) family. Apoptosis inducing factor (AIF) and endonuclease G translocate to the nucleus and participate in DNA degradation [186].

Several mechanisms have been implicated in MnSOD protection from apoptosis. MnSOD overexpression protects from mitochondrial dysfunction and loss of mitochondrial membrane potential caused by various agents that induce apoptosis, including ionizing radiation [187], as well as Fe(II), amyloid β-peptide, NO generating agents [188], and tumor necrosis factor-related apoptosis-inducing ligand (TRAIL) [189]. MnSOD also prevents the release of various proteins from mitochondria that carry out apoptosis, such as cytochrome c [187] and Smac/DIABLO [189].

Modulation of ROS levels, and corresponding ROS-mediated damage, is another mechanism of MnSOD suppression of apoptosis. Overexpression of MnSOD protects FsaII murine fibrosarcoma cells from apoptosis caused by electron transport inhibitors antimycin and rotenone by inhibiting caspase 3 activity and poly(ADP-ribose) polymerase cleavage, in part, by altering mitochondrial ROS levels [190]. In a study by Keller *et al*. [188], overexpression of MnSOD protects PC6 pheochromocytoma cells from apoptosis induced by Fe(II), amyloid β-peptide, and NO generating agents by inhibiting peroxynitrite production and lipid peroxidation, as well as prevents mitochondrial transmembrane potential collapse and a decrease in mitochondrial activity caused by these agents. MnSOD also protects against apoptosis induced by ionizing radiation and exposure to different anticancer agents [191].

MnSOD also affects apoptosis stimulated by inflammatory cytokines. Hirose *et al*. found that in A375 human melanoma cells and Chinese hamster ovary cells, overexpression of MnSOD protects cells from the toxic effects of inflammatory cytokines [191]. Tumor necrosis factor (TNF) induces apoptosis, in part, by stimulating mitochondrial ROS production [192]. MnSOD expression is stimulated by TNF and may act as an adaptive response to protect cells from further exposure to TNF [193]. As a demonstration of this concept, overexpression of MnSOD confers protection of a variety of cell lines against apoptosis induced by tumor necrosis factor (TNF) [194]. This adaptive response may protect cells from apoptosis caused by other toxic agents. Pretreatment of hippocampal cells with TNF-α protects the cells from apoptosis induced by Fe(II) and amyloid β-peptide, in part, by stimulating MnSOD expression [195]. Work by this laboratory demonstrated that tamoxifen, the antiestrogen used to treat breast cancer, enhances TNF-α-induced expression of MnSOD by increasing the binding of the p50/p65 heterodimer of NF-κB to an enhancer in the second intron of the MnSOD gene [196]. Further work showed that stimulation of MnSOD expression is part of the mechanism of tamoxifen-mediated protection from adriamycin-induced apoptosis in cardiac tissue [197]. Pardo *et al*. found that in human Jurkat T cells, caspase-dependent degradation of MnSOD is important for Fas receptor-mediated apoptosis, resulting in an increase in superoxide production and enhanced apoptosis [198]. A mechanism of MnSOD-induced resistance to TNF-α is thought to be an increase in the steady-state levels of hydrogen peroxide [199].

### 4.5. Mitochondrial Control of Innate and Adaptive Immunity

Innate immunity is the first line of defense for cells against the onslaught of foreign microbes or cellular damage. Innate immunity is carried out by a series of pattern recognition receptors (PRRs) that recognize key components of microbial invaders referred to as pathogen-associated molecular patterns (PAMPs) or various endogenous cellular components that mark cells as damaged or injured known as danger-associated molecular patterns (DAMPs). The PRRs can be membrane bound, such as the Toll-like receptors found at the membranes of endosomes, lysosomes, or the cell surface. PRRs can also be cytosolic, such as the nucleotide-binding oligomerization domain (NOD)-like receptors (NLRs) and the retinoic acid-inducible gene-1 (RIG-1)-like receptors (RLRs) [200–202]. Activation of PRRs leads to activation of several transcription factors (NF-κB, AP-1, interferon-regulatory factor (IRF)) [202]. PRR activation can also cause formation of inflammasomes, multiprotein complexes consisting of a sensing protein (NLR), an adaptor protein (apoptosis-associated speck-like protein containing a CARD (ASC)), and a caspase, such as caspase 1 [203]. The inflammasome is a platform for activation of various inflammatory cytokines, including interleukin-(IL) 1β, in response to the presence of PAMPs or DAMPs [202].

Mitochondria are important for the initiation of immune responses. Seth *et al*. identified a mitochondrial antiviral signaling (MAVS) protein important for NF-κB and IRF-3-dependent expression of interferon-β (IFN-β) in response to viral infection. Silencing MAVS expression by RNAi or overexpression of MAVS abolishes or enhances IFN-β expression, respectively. Mitochondrial localization of MAVS is essential for its function, since localization of MAVS to either the endoplasmic reticulum or plasma membrane greatly reduces the ability of MAVS to induce IFN-β expression [204]. Zhan *et al*. discovered that injury-induced release of mitochondrial DNA or formyl peptides activates polymorphonuclear neutrophils and can lead to neutrophil-mediated organ injury [205]. Mitochondrial DNA is also important for activation of innate immunity. In J774A.1 macrophages, treatment with ethidium bromide to deplete the cells of mitochondrial DNA (ρ^0^ cells) inhibits LPS- and ATP-induced caspase-1 activation and IL-1β secretion. Cytosolic release of mitochondrial DNA is also important for inflammasome activation, as treatment with DNase I inhibits LPS- and ATP-induced activation of the inflammasome, while transfection with mtDNA enhances this effect in bone marrow-derived macrophages [206].

ROS are vital for inflammasome activation (reviewed in [207]). Dostert *et al.* found that NADPH oxidase (Nox)-generated ROS are involved in asbestos-induced Nalp3 inflammasome activation and subsequent interleukin-1β (IL-1β) secretion in human macrophages. Inhibition of Nox activity using diphenylene iodonium chloride or apocynin or scavenging of ROS using *N*-acetylcysteine or (2*R*,4*R*)-4-aminopyrrolidine-2,4-dicarboxylate (APDC) hinders asbestos-induced IL-1β activation [208]. Later work by Meissner *et al*. revealed that caspase-1 activation and production of IL-1β occurs in mononuclear phagocytes that lack active Nox [209], suggesting other sources of ROS may be involved in inflammasome activation.

Recent work by two laboratories has revealed that mitochondria are the major source of ROS involved in the activation of inflammasomes. Using the THP1 macrophage cell line, Zhou *et al*. found that chemical inhibition of various components of the electron transport chain increases mitochondrial ROS formation and correlates with an increase in activated IL-1β. Inflammasome components NLRP3 and ASC localize to endoplasmic reticulum and mitochondria in response to inflammasome-stimulating agents MSU, alum, and nigericin. The authors demonstrate the importance of VDAC in mitochondrial ROS-dependent inflammasome formation. Knockdown of VDAC1 and VDAC2 expression or overexpression of Bcl-2 (which inhibits VDAC function) inhibit caspase-1 activation and formation of mature IL-1β [210]. In LPS or ATP-stimulated macrophages, treatment with rotenone increases caspase-1 activation and IL-1β secretion, which is abolished with the antioxidant Mito-TEMPO [206].

Mitochondrial ROS can also activate proinflammatory cytokines independent of inflammasome formation. Bulua *et al*., using mouse embryonic fibroblasts expressing different mutations of the type 1 TNF receptor (TNFR1) associated with the autoinflammatory disorder tumor necrosis factor receptorassociated periodic syndrome (TRAPS), found that TNFR1 mutant cells have elevated basal levels of mitochondrial ROS, and this mitochondrial ROS is important for lipopolysaccharide-stimulated production of the proinflammatory cytokines IL-6 and TNF, but not IL-1β, in the absence of inflammasome formation. Scavenging of mitochondrial ROS inhibited LPS-dependent cytokine production [211].

Zhou *et al*. and Nakahira *et al*. also link autophagy with inflammasome activation. Inhibition of autophagy using the chemical inhibitor 3-methyladenine (3-MA) [210] or knockdown of LC3 [206] or beclin-1 [206,210] causes increased mitochondrial ROS formation and a corresponding increase in caspase-1 activation and IL-1β secretion. Inhibition of autophagy also prevents LPS and ATP-stimulation of mtDNA release into the cytosol, correlating with a decrease in caspase-1 activation [206]. The results of these studies suggest a role for autophagy for the removal of damaged mitochondria, which prevents the activation of innate immunity through inflammasome formation. When autophagy is inhibited, damaged mitochondria accumulate, resulting in increased basal levels of mitochondrial ROS and increased inflammasome formation. These studies also suggest exciting possibilities for mitochondrial antioxidant enzymes, particularly MnSOD, in the regulation of innate immunity and inflammatory diseases through both inflammasome-dependent and -independent mechanisms.

ROS production is also vital for adaptive immunity and can affect T cell function. ROS are important for T cell activation by promoting lipid raft formation for assembly of the T cell activation complex [212]. Moulian *et al.* report that peroxynitrite formation in the thymus is associated with apoptosis in human thymocytes [213]. Glutathione depletion is an important mechanism for activated T cell death induced by the tryptophan metabolite 3-hydroxyanthranilic acid [214]. ROS are essential for hematopoietic progenitor cell differentiation in *Drosophila* [215]. However, little is known of the role of MnSOD in adaptive immunity. Using a thymus-specific MnSOD knockout mouse model, Case *et al.* [216] demonstrated that elevation of superoxide negatively impacts T-cell development and alters adaptive immune system function by increasing apoptosis and developmental defects in the T-cell population. Thymus-specific knockout of MnSOD leads to increased immunodeficiency and greater susceptibility to influenza A H1N1 infection compared to control mice, which were rescued by treatment with superoxide scavengers Tempol or CTPO. These results reveal the importance of MnSOD in maintaining adaptive immune response and may lead to therapies for various immunological disorders involving T-cell dysfunction.

### 4.6. Mitochondrial DNA (mtDNA) Stability

mtDNA is a circular double-stranded DNA molecule that is 16,569 bp long and encodes 37 genes. These genes encode for 22 tRNAs and 2 rRNAs that are essential for translation of the remaining 13 genes, which comprise various components of the electron transport chain [217]. The remainder of proteins found in mitochondria are encoded by nuclear DNA, synthesized in the cytosol, and imported in mitochondria. mtDNA is organized into discrete foci called nucleoids containing 6–10 individual mtDNA genomes each [218,219]. Nucleoids also contain a number of proteins important for synthesis and transcription of mtDNA, including mitochondrial single-strand DNA binding protein (mtSSB), mitochondrial transcription factor A, and mitochondrial DNA polymerase gamma (Polγ) [219–221]. Mitochondrial function decreases with age [222] and is associated with both a decrease in mtDNA content and an increase in oxidative mtDNA damage [223]. As a result, mtDNA damage is linked to numerous age-related conditions [224], including heart failure, Parkinson’s disease, diabetes [225], and cancer [226,227].

mtDNA is susceptible to damage induced by a variety of agents, including ultraviolet [228] and ionizing radiation [229], as well as ROS [127,228,229]. Mambo *et al.* indentified the D-loop as a region of mtDNA that is highly susceptible to oxidative damage compared to other regions [230]. Cortopassi and Wang found that mtDNA sequences that encode various Complex I subunits are statistically more susceptible to damage, which may contribute to increased superoxide production and age-related diseases [231]. In cells that lack mtDNA (ρ^0^ cells) or cells that express mtDNA with a common 4977-bp deletion, there is a significant increase in ROS production compared to either parental cells or cells in which wild-type mtDNA has been introduced [232], and MnSOD is expressed as an adaptive response to the depletion of mtDNA [233]. A vicious cycle is set in motion in which mtDNA damage results in altered mitochondrial function, leading to increased ROS production, which causes more mtDNA damage [234]. Using simian virus 40 (SV40)-transformed human fibroblast cell line GM00637E, Yakes and van Houten found that mtDNA is more sensitive to oxidative stress-induced damage caused by hydrogen peroxide treatment than nuclear DNA, mtDNA damage occurs more rapidly, and during prolonged exposure to hydrogen peroxide (60 min), mtDNA damage was not repaired. mtDNA damage in GM00637E cells correlates with a decrease in mitochondrial function as measured by MTT reduction [235]. Hydrogen peroxide is identified as an important mediator of oxidative mtDNA damage induced by UV irradiation [228].

Polγ is susceptible to oxidative stress-mediated inactivation, which may affect mtDNA replication and repair. For example, Graziewicz *et al*. found that treatment of the catalytic subunit of Polγ with hydrogen peroxide causes oxidative modification of the protein and results in an approximately 50% reduction in polymerase activity in a time- and dose-dependent manner, as well as a decrease in Polγ DNA binding activity. Polγ is more sensitive to oxidative inactivation than Polβ and Polα, two nuclear DNA polymerases.

MnSOD plays an important role in protecting mtDNA from ROS-induced damage. Steinman *et al.* found that MnSOD associates with DNA in *Escherichia coli* K-12 cells [236]. Oxidative mtDNA damage increases with age in mice, but this damage is much greater in the livers of MnSOD heterozygous knockout mice compared to wild-type controls [103]. MnSOD expression is increased in peripheral blood mononuclear cells from type 2 diabetic patients as an adaptive response to increased oxidative mtDNA damage in these cells [237]. In bovine retina endothelial cells, overexpression of MnSOD or treatment with a MnSOD mimetic inhibits oxidative mtDNA damage caused by high glucose, resulting in an increase in the expression of various electron transport chain components compared to cells treated with glucose alone [238]. Overexpression of MnSOD also protects mtDNA from oxidative damage induced by exposure to UV radiation [228], as well as mtDNA damage and depletion caused by acute ethanol exposure [129,239]. MnSOD has been identified as part of the nucleoid complex, where it interacts with mtDNA, Polγ, and glutathione peroxidase and may play a role in protecting mtDNA from oxidative stress-induced damage [240]. Work by this laboratory confirmed that MnSOD interacts with Polγ and mtDNA, and demonstrated that MnSOD may be important for protecting mtDNA from UV-induced damage by preventing inactivation of Polγ [241]. These studies suggest a potential role for MnSOD in the prevention or treatment of diseases associated with mtDNA damage.

### 4.7. Cellular Lipid Integrity

Lipids, especially polyunsaturated lipids, are susceptible to attack by ROS. A variety of compounds resulting from lipid peroxidation have been identified, such as 4-hydroxynonenal and malondialdehyde, as well as products derived from the oxidative attack of cholesterol, cholesterol esters, and sphingholipids [242]. Lipids are also vulnerable to attack by RNS to form different oxidation and nitration products [243–246], and these RNS-derived lipid peroxidation products may have importance in such diverse functions as inflammation [243] and ischemia/reperfusion injury [247].

Altered MnSOD expression or activity can also have consequences on total cellular, and mitochondrial, lipid integrity. Heterozygous MnSOD knockout mice have substantially more lipid peroxidation (as determined by immunohistological staining for 8-isoprostane, a marker of lipid peroxidation) at day 10 after birth compared to wild-type counterparts [248]. On the contrary, overexpression of MnSOD in the PC6 pheochromocytoma cell line attenuates lipid peroxidation and the formation 4-HNE-adducted proteins caused by treatment with Fe(II), sodium nitroprusside (a nitric oxide-generating agent), and amyloid β-peptide, as well as lipid peroxidation *in vivo* caused by local cerebral ischemia [188]. Administration of MnSOD plasmid/liposome intraesophageally or intraorally into mice to induce localized overexpression of MnSOD protects the animals from lipid peroxidation induced by ionizing radiation compared to control mice [249].MnSOD may also play a role in lipid peroxidation caused by hypertension. Using normotensive rats and either spontaneously hypertensive or induced hypertensive rats, Ohtsuki *et al.* discovered that lipid peroxidation in enriched mitochondrial fractions of brain tissue was greater with age in spontaneously hypertensive and deoxycorticosterone acetate (DOCA)-induced hypertensive rats compared to normotensive or vehicle-treated rats, respectively. No significant changes in mitochondrial lipid peroxidation were observed in either heart or kidney tissue. MnSOD expression increased with age only in the normotensive rats in brain tissue. These results suggest that altered superoxide radical metabolism may contribute to neurological conditions associated with hypertension [250].

Since MnSOD contains manganese in its active site [85], factors that affect manganese availability can also have a marked effect on lipid peroxidation. Manganese deficiency significantly reduces age-related increases in MnSOD activity compared to manganese-sufficient rats, correlating with a 5-fold increase in lipid peroxidation in manganese-deficient rat liver compared to a 3-fold increase in rats receiving adequate amounts of manganese [251]. Malecki and Greger found that lipid peroxidation was greater in rat heart mitochondria in animals deficient in Mn compared to rats receiving adequate amounts of manganese, which inversely correlated with MnSOD activity [252]. Manganese deficiency has been identified in patients with sundry diseases, including epilepsy [253], diabetes mellitus [254], and patients receiving hemodialysis [255]. Future studies of how this manganese deficiency correlates with MnSOD activity and lipid peroxidation in these, and other, diseases may provide important insights into mechanisms of disease development.

Cardiolipin is a tetra-acylated glycerophospholipid composed of two phosphotidyl groups linked by glycerol, resulting in a lipid with four hydrocarbon chains and three chiral centers [256] Cardiolipin is found almost entirely in the inner mitochondrial membrane [257]. Altered levels and composition of cardiolipin is associated with various diseases involving mitochondrial dysfunction, including ischemia/reperfusion injury, diabetes, heart failure [258,259], aging [260,261], and cancer [262–264]. Cardiolipin is also linked to Barth syndrome, an X-linked cardiomyopathic disease characterized by neutropenia, cardiomyopathy, and 3-methylglutaconic aciduria [265]. Barth syndrome is caused, in part, by a mutation in tafazzin, a phospholipid acyltransferase important for assembly of cardiolipin [266–268].

Cardiolipin is vital for the proper function of many mitochondrial activities. For example, cardiolipin is important for the initiation of apoptosis [269–275]. Peroxidation of cardiolipin leads to the release of cytochrome *c* from mitochondria [270,276] and opening of the permeability transition pore (PTP) by inactivation of the adenine nucleotide translocator (ANT) [270]. Cardiolipin is vital for the activities of individual components of the electron transport chain [277] and the assembly of these components into supramolecular complexes [278,279]. Cardiolipin is susceptible to peroxidation by ROS [261,280,281], and ROS-mediated inactivation of different complexes of the electron transport chain parallel oxidative damage of cardiolipin. Treatment with a combination of SOD and catalase, or supplementation with exogenous cardiolipin, reverses the effects of ROS on electron transport chain components and cardiolipin peroxidation [282–286]. In HaCaT human keratinocytes, low dose UVB radiation leads to decreased activities of different electron transport chain components with a concurrent decrease in cardiolipin content. MnSOD protein levels increase after UVB exposure as an adaptive response to UVB exposure [287]. These studies imply a potential mechanism for MnSOD in protecting mitochondria from ROS-induced damage by preventing cardiolipin peroxidation.

## 5. Implications for Disease

### 5.1. Cancer

ROS are important for the development of cancer by many mechanisms, including ROS-mediated DNA damage, activation of various transcription factors, and initiation of multiple signal transduction pathways [288–290]. MnSOD seems to play a dual role in cancer development and progression (recently reviewed in [291]). Some studies show elevated MnSOD expression in cancer compared to normal tissue [292–298], while other studies show MnSOD is reduced in many types of cancer [28], including breast cancer [299,300], pancreatic cancer [301], and ovarian cancer [302]. Depending on the type of cancer and its stage of development, MnSOD may act as either a tumor suppressor or a tumor promoter. For example, MnSOD expression is associated with an increase in lymph node metastases of both colorectal [303] and gastric cancer [292], and is important for the metastatic behavior of the estrogen-independent breast cancer cell line MDA-MB-231 [304], as well as progestin-stimulation of migration and invasion in T47D human breast cancer cells [305]. One mechanism of MnSOD-enhanced metastasis is stimulation of matrix metalloproteinase expression through hydrogen peroxide-dependent mechanisms [306,307]. On the other hand, other studies have demonstrated that overexpression of MnSOD inhibits many of the hallmark properties of cancer, such as increased growth rate, invasiveness, and anchorage independent cell growth [300,308–311].

Alteration of ROS levels induced by chemical carcinogens is important for the tumor suppressive effects of MnSOD [312–314]. Overexpression of MnSOD sensitizes cancer cells to cell death induced by various ROS-generating agents both *in vitro* and *in vivo* [315]. In HEK293 human embryonic kidney cells, overexpression of an active site mutant of MnSOD lacking product inhibition causes ROS-mediated growth retardation, which was abrogated by co-expression of catalase [316]. Ridnour *et al*. discovered that MnSOD overexpression in XR23M transformed x-ray immortalized rat embryo fibroblast cells decreases the tumorigenicity of these cells (decreased colony formation, diminished tumor growth *in vivo*, and diminution of metastatic potential) in a manner that correlates with MnSOD-mediated production of hydrogen peroxide [312]. MnSOD overexpression in PC-3 human prostate cancer cells have higher levels of hydrogen peroxide and mitochondrial membrane potential compared to parental cells, correlating with decreased cell growth [309].

A major concentration of study for this laboratory has been to refine the importance of MnSOD in oxidative stress-induced tumor initiation and promotion. In the Fsa-II mouse fibrosarcoma cell line, MnSOD overexpression decreases proliferation and colony formation with a simultaneous induction of differentiation, in part by inhibiting AP-1 DNA binding activity and expression of AP-1 target genes [317]. MnSOD overexpression both inhibits 5-azacytidine-induced apoptosis and enhances differentiation in Fsa-II cells by activation of NF-κB and the ERK MAP kinase pathway [318]. Using the DMBA (7,12-dimethylbenz[*a*]-anthracene)/TPA (12-*O*-tetradecanoylphorbol-13-acetate) treatment regimen for tumor initiation and promotion, this laboratory found that overexpression of MnSOD reduces the incidence and number of papillomas per animal compared to non-transgenic mice by inhibiting TPA-induced oxidative stress [319].

Based on the results in MnSOD overexpressing mice, it seems logical that knockdown of MnSOD would enhance DMBA/TPA-induced tumorigenesis. DMBA/TPA treatment of heterozygous knockout of MnSOD in the C57BL/6 mouse model resulted in a similar number of papillomas as wildtype mice. The similarity in papilloma formation between the two genotypes results from an increase in both proliferation and apoptosis in the basal layer of the epidermis. A major difference between the MnSOD knockout and wildtype mice is an increase in oxidative stress, signified by an increase in oxidized proteins [320]. It was later revealed that apoptosis precedes proliferation in the basal layer of the epidermis. Apoptosis peaks at 6 h post-TPA treatment, while mitosis peaks 24 h after treatment, and proliferation is less in wildtype mice than MnSOD knockout animals. When mice were treated with the MnSOD mimetic MnTE-2-PyP5+ 12 h subsequent to each TPA treatment, no effect on apoptosis was observed, but a significant reduction in TPA-stimulated proliferation and oxidative modification of proteins occurred, resulting in a 50% reduction in tumor incidence compared to DMBA/TPA treatment alone. These results suggest that oxidative stress is an important event early in tumorigenesis and provide a potential mechanism by which MnSOD inhibits cancer formation [321].

Another area of research for MnSOD in cancer development is the effect of single nucleotide polymorphisms (SNPs) in the SOD2 gene that alter the overall expression, function, or subcellular localization of MnSOD. One such polymorphism, resulting in a C→T transition, causes the conversion of valine to alanine at amino acid 16 in the mitochondrial signaling sequence of MnSOD [322]. The Ala16 variant can easily enter the matrix and has higher activity than the Val16 variant, which remains embedded in the inner membrane. Differences in the secondary structure of the two variants explain the difference in mitochondrial migration. The Ala16 variant has a partial α-helical structure, while the Val16 variant adopts a β-sheet structure. The structural differences between the Ala16 and Val16 variants of MnSOD affect the interaction of the protein with the Tim23 import channel in the inner mitochondrial membrane, which affect localization of MnSOD within the matrix and subsequent activity [323,324].

The specific role of the Val16Ala SNP in cancer development remains controversial [325]. Some studies suggest a link between the Val16Ala SNP and risk of ovarian cancer [326,327] and lung cancer [328]. Individuals with the Val/Val genotype have a significantly greater risk of lung cancer compared to those with the Ala/Ala genotype [329,330]. The Val/Val and Val/Ala genotypes increase lung cancer risk in an age-dependent manner, with patients younger than 55 years carrying either the Val/Val or Val/Ala genotype having a greater risk than older patients [330]. The opposite trend appears with prostate cancer. Men that are homozygous for the Ala polymorphism have a 70% increased prostate cancer risk than men homozygous for the Val polymorphism [331], and there is an increased risk for early-onset prostate cancer (≤65 years) in men that are homozygous or heterozygous for the Ala polymorphism compared to Val homozygous men [332].

Many other polymorphisms have been identified in the SOD2 gene. Ile58 is an important amino acid that lies at the tetrameric interface of the four monomers that comprise mature MnSOD protein [81]. The Ile58Thr polymorphism (rs1141718) causes destabilization of the tetrameric interface and formation of primarily dimeric MnSOD. The thermal sensitivity of MnSOD is also increased with the Ile58Thr polymorphism. Borgstahl *et al*. found that Thr58 variant of MnSOD had about half of the enzyme activity as the normal protein at all temperatures tested [333]. The diminished enzyme activity of the Thr58 variant of MnSOD reduces its tumor suppressor activity in MCF-7 human breast cancer cells compared to the Ile58 variant [334]. Other polymorphisms of MnSOD that have been identified include Leu60Phe (rs11575993) [335] and G1677T (rs2Y758Y339) [336]. Leu60Phe is observed in the Jurkat human T-cell leukemia cell line, resulting in reduced MnSOD enzyme activity in Jurkat cells compared to normal human peripheral blood lymphocytes [335]. The G1677T polymorphism generates a potential glucocorticoid receptor binding site near the enhancer localized within the second intron of the SOD2 gene. The T/T genotype correlates with a reduction in the risk of lung cancer [336].

MnSOD polymorphisms, while affecting cancer incidence, may also have an effect on response to treatment. Using patient populations from the United States and Norway, Glynn *et al.* found that patients with the Ala allele for the Val16Ala SNP, and were receiving cyclophosphamide chemotherapy regimens had a significant reduction in survival compared to patients with the Val allele [337]. Yao *et al*. studied the effects of the Val16Ala MnSOD polymorphism on treatment-induced hematological toxicity and disease-free survival using data from a Southwest Oncology Group (SWOG) clinical trial (S8897). Breast cancer patients receiving adjuvant therapy (either the combination of cyclophosphamide, doxorubicin, and 5-fluorouracil or the combination of cyclophosphamide, methotrexate, and 5-fluorouracil) that were homozygous for cytosine allele and the Val16Ala polymorphism had a significant reduction in the risk of developing grade 3 and 4 neutropenia than women homozygous for the thymidine allele. The homozygous cytosine allele group also had a non-significant reduced risk of developing grade 3 and 4 leucopenia. Interestingly, women that were heterozygous for the cytosine allele had a non-significant increased risk of disease recurrence or death [338]. These results suggest a need for genotyping patients for MnSOD SNPs to tailor the cancer chemotherapy regime to improve patient survival and to minimize side effects.

One of the hallmarks of cancer is the referred to as the Warburg effect, a process by which cancer cells switch from oxidative phosphorylation for ATP production to glycolysis-dependent ATP production, even at high levels of oxygen [339], due to dysfunctional oxidative phosphorylation in mitochondria caused by various mechanisms, including damage to mtDNA and nuclear DNA that encodes various genes for oxidative phosphorylation [340,341]. Genes encoding various enzymes in the glycolysis pathway are overexpressed in numerous cancer types [342]. The switch to glycolysis gives cancer cells several advantages, including the production of various substrates essential for proliferation [343] and protection from ROS-mediated apoptosis [344]. Since cancer cells develop a “sweet tooth” for glucose, various strategies targeting the glycolysis pathway have been developed to treat cancer [343,345].

Sirtuin 3 (SIRT3) is a NAD^+^-dependent deacetylase localized to mitochondria [346]. SIRT3 is an important tumor suppressor that helps to preserve mitochondrial integrity, inhibits mitochondrial ROS production, and hinders the Warburg effect by suppressing ROS-dependent stabilization of HIF-1α and expression of HIF-1α target genes [347–349]. MnSOD can undergo various post-translational modifications that alters MnSOD enzyme activity (reviewed in [350]). MnSOD has recently been identified as a SIRT3 target, with SIRT3-dependent deacetylation of MnSOD at Lys122 being vital for maintaining MnSOD in an active state [351], and regulation of MnSOD activity by SIRT3 may be important in the cellular response to nutrient status and various types of stress (reviewed in [352]).

Because of the importance of MnSOD in protecting different mitochondria-centered metabolic enzymes from ROS-mediated deactivation, the decrease in MnSOD expression and activity associated with cancer, and the fact MnSOD is a target of SIRT3 deacetylase, it is tempting to speculate that MnSOD may be essential for initiation of the Warburg effect in the development and progression of cancer. Future work may need to be focused on the link between MnSOD and the Warburg effect. Other work may also be concentrated on which comes first, decreased MnSOD expression or the Warburg effect, and whether a feedback mechanism exists between MnSOD-dependent ROS detoxification and the metabolic switch to glycolysis.

### 5.2. Cardiovascular Disease

Altered expression of MnSOD is observed in various cardiovascular disorders. In ventricular myocardium from heart failure patients, there is a significant increase in superoxide production, as determined by electron paramagnetic resonance, compared to non-failing heart tissue. While MnSOD mRNA levels are elevated in failing myocardium, there is a decrease in protein levels and enzyme activity compared to non-failing heart, suggesting that some post-transcriptional regulation or post-translational modification of MnSOD occurs to prevent a complete adaptive response to the increased superoxide levels [353]. Myocardial infarction in rat heart causes a region-specific expression of MnSOD. The left ventricle shows an early increase in MnSOD mRNA levels at 6 h post-infarction, peaks at 12 h, then declines at 48 h. On the other hand, the right ventricle shows a steady increase in MnSOD mRNA up to 48 h. The increase in MnSOD mRNA does not lead, however, to a corresponding increase in enzyme activity [354]. Csonka *et al.* found that expression of glutathione peroxidase, CuZnSOD, and MnSOD are elevated in spontaneously hypertensive (SH) rats compared to controls, and this elevation in antioxidant capacity protects the SH rats from further oxidative stress caused by infusion of hydrogen peroxide [355]. Khaper *et al*. followed the expression of various antioxidant enzymes, including MnSOD, at different times following myocardial infarction in rats, and found that MnSOD mRNA levels were reduced early (1 week) and long after (16 weeks) infarction, but MnSOD mRNA was near control levels at 4 weeks post-infarction [356].

MnSOD knockout animal models have proven valuable in determining the mechanisms underlying changes in cardiac function with variations in MnSOD expression. Using a mouse model with tetracycline-dependent expression of MnSOD, Loch *et al*. found that loss of MnSOD results in reduced left ventricular function (decreased fraction shortening and ejection fraction, increased left ventricular diameter at systole). These mice also developed heart hypertrophy [357]. Reduced MnSOD levels also increase the sensitivity of cardiomyocytes to mitochondria-centered apoptosis induced by different agents [358]. MnSOD is also important for blood pressure regulation. Decreased expression of MnSOD in various mouse models results in impaired vasorelaxation [359,360] and greater vasoconstriction [360] induced by various agents in old animals compared to young animals, which show no significant differences in responses.

Not only are polymorphisms of the MnSOD gene important for cancer development, they also have an impact on cardiovascular disease. Kakko *et al.* found that the Val16 polymorphism of mitochondrial signaling sequence of MnSOD was a minor component explaining differences in intima-media thickness (a measure of carotic atherosclerosis) between subjects [361]. The Val/Val genotype is an independent risk factor for coronary artery disease (CAD) and the frequency of this allele is significantly higher in CAD patients that had an acute myocardial infarction [362], and is also higher in patients with vasospastic angina compared to healthy subjects [363].

MnSOD is vital for protecting cardiac tissue from damage caused by various factors. Miller *et al*. identified MnSOD as an important component in protecting endothelial cells from cyclooxygenase-1 (COX-1)-mediated dysfunction [364]. Negoro *et al.* identified MnSOD as a STAT3 (signal transducer and activator of transcription 3) target gene vital for STAT3-mediated protection of cardiac tissue from hypoxia/reoxygenation-induced injury [365]. Jin *et al.* discovered that after ischemia/reperfusion of mouse heart, there is a significant increase in cytosolic MnSOD and release of cytochrome *c* from mitochondria, which was prevented by ischemic preconditioning or treatment with the mitochondria permeability transition pore inhibitor cyclosporin A [366].

Nitration is an important post-translational modification of MnSOD that can alter the cellular response to normal and stress-induced conditions. MacMillan-Crow *et al*. identified MnSOD as a protein nitrated in chronically rejected kidney transplants, correlating with a decrease in MnSOD enzyme activity in these transplanted organs. Exposure of recombinant MnSOD to peroxynitrite *in vitro* results in a statistically significant decrease in MnSOD enzyme activity, confirming the importance of nitration in modulating MnSOD activity [367]. The site of nitration on MnSOD was later identified as Tyr34 [368,369], which is found in the active site on MnSOD. X-ray crystallographic studies of both the unmodified and Tyr34-nitrated MnSOD reveal that nitration at Tyr34 can weaken the hydrogen bonding of the active site, which may affect proton transfer during dismutation of superoxide, as well as impede substrate access and binding to the active site [370].

MnSOD nitration can have significant effects on cardiovascular disease development and progression caused by therapeutic agents. Xu *et al.* identified Tyr34-nitrated MnSOD in atherosclerotic cardiac atrium from diabetic patients [371]. Cyclosporine A (CsA), while an important immunosuppressant used in organ transplant patients, also has significant side effects, including cardiovascular injury [372]. Oxidative stress appears to be a component of CsA-mediated injury [373]. CsA stimulates peroxynitrite production and subsequent nitration of many proteins in both aortic endothelium and bovine aortic endothelial cells, including MnSOD [374]. Cyclosporine A-induced nitration and inactivation of MnSOD is dependent, in part, on endothelial nitric oxide synthase (eNOS)-dependent production of nitric oxide and mitochondrial superoxide [375].

### 5.3. Neurological Disorders

Not only does MnSOD expression affect cardiovascular disease, but it also appears to have crucial consequences on neurodegenerative disorders, including ischemia/reperfusion injury caused by stroke [376], sleep apnea-induced chronic intermittent hypoxia [377], HIV-associated dementia [378], and neurological dysfunction resulting from traumatic brain injury [379,380]. In cultured neuronal cells, MnSOD transfection prevents iron- and β-amyloid-induced cell death [188], while knockdown of MnSOD augments glutamate-induced toxicity in mouse cortical neurons [381]. Overexpression of MnSOD protects cortical cultures from *N*-methyl-d-aspartate (NMDA) and nitric oxide-induced toxicity [382], as well as decreases lesion volume after traumatic brain injury compared to wildtype mouse littermates [383]. Homozygous knockout mice have death of neurons in both the basal ganglia and the brain stem and show severe mitochondrial damage [106]. In *Sod2*^−^*^/^*^−^ B6D2F1 mice, ataxia develops by postnatal day 11 (P11) and frequent seizures occur by P14, which are associated with damage to the thalamus, motor cortex, and the brain stem (olivary nucleus, vestibular nucleus, motor trigeminal nucleus, and mesencephalic trigeminal nucleus) at P11–13, which becomes more extensive by P15–16. Vacuole formation occurs in the pyramidal layer of the hippocampus, different regions of the thalamus and brain stem, as well as the deeper layers of the motor cortex [384]. Vacuole formation is also observed in the brain of a *Drosophila* model with reduced MnSOD expression, resulting in neuronal apoptosis and a diminution of normal olfactory behavior [102].

MnSOD appears to be important for the development and progression of Alzheimer’s disease (AD). In AD patients, there is a significant increase in MnSOD expression in the C1, CA2/3, and C4 regions of the hippocampus compared to non-AD patients. These results suggest that MnSOD expression is part of a compensatory mechanism in the hippocampus for the increase in ROS linked to AD progression [385]. MnSOD overexpression in the Tg19959 mouse model of AD has no effect on amyloid precursor protein expression or processing, but does cause a significant decrease in amyloid plaque burden in the cortex and a nonsignificant decrease of plaques in the hippocampus and a decrease in cortical microglia. These mice also show improved spatial memory retention [386]. On the other hand, heterozygous knock-out of MnSOD in T19959 mice increases the oxidative damage to various brain proteins and results in a significant increase in plaque burden in the hippocampus, retrospenial/motor cortex, and cortex [387].

Much work by this laboratory has focused on the role of MnSOD in the development and progression of AD. Using isolated primary neurons from wild-type or amyloid precursor protein (APP) and presenilin (PS1) double knock-in (APP/PS1) mice, Sompol *et al.* discovered that mature APP/PS1 neurons have decreased MnSOD expression and an increase in the colocalization of nitrotyrosine with MnSOD, suggesting that MnSOD enzyme activity may be inhibited by tyrosine nitration [388]. Increased MnSOD nitration was observed in APP/PS1 mice compared to wild-type controls, correlating with both a decrease in MnSOD enzyme activity and reduced mitochondrial activity [389], suggesting that tyrosine nitration of MnSOD may be an important component of neuronal injury in AD.

The interaction of MnSOD with various proteins can have dramatic effects on MnSOD activity and, ultimately, on global cellular homeostasis. Eldridge *et al*. used a gel-based mass spectrometry technique for identifying proteins that interact with MnSOD that enhance MnSOD enzyme activity during adaptive radioresistance. These proteins are involved in such diverse functions as DNA repair, apoptosis, cell cycle regulation, and mitochondrial function [390]. The cell cycle regulators cyclin D1 and cdk4 have been identified as proteins that directly interact with MnSOD. This interaction may be important for the regulation of mitochondrial function during low dose radiation-induced adaptive response [391]. Studies like this may provide important insights into proteins that interact with MnSOD and the role of these interactions in regulating mitochondrial function, in particular, and the overall affect of these interactions on global cellular function and disease development.

Off-target effects of chemotherapeutic drugs are a major research area for this laboratory, and recent work in this laboratory demonstrates the importance of MnSOD/protein interactions. One such avenue of study is the cognitive decline associated with different chemotherapy regimens referred to as “chemobrain” (see Effects of MnSOD on Mitochondrial Integrity/Function above). Using the SK-N-SH human neuroblastoma cell line, this laboratory showed that treatment with the cancer chemotherapy drug paclitaxel (PTX) causes an increase in MnSOD expression with no corresponding increase in MnSOD enzyme activity, which correlated with an increase in mitochondrial localization of p53. p53 interacts directly with MnSOD, and chemical inhibition of PTX-induced mitochondrial translocation of p53 using pifithrin-μ partially rescues MnSOD enzyme activity from inhibition induced by PTX. These results suggest that inhibition of MnSOD enzyme activity by direct interaction of MnSOD with p53 may be an important mechanism of PTX-induced neurotoxicity [392].

Parkinson’s disease (PD) is a progressive neurodegenerative disorder marked by the loss of dopaminergic neurons in the substantia nigra, basal nuclei, and the tectum mesencephalicum, as well as the formation of eosinophilic fibrillary intracellular inclusions in neurons referred to as Lewy bodies [393]. Altered mitochondrial function and dynamics are important characteristics for the progression of PD and have been linked to several PD-specific genes [394–396]. Mitochondrial ROS are a key component to PD development [397], with complex I of the electron transport chain acting as both a victim, and source, of ROS [122,125,397]. Several studies demonstrate that while polymorphisms of the MnSOD gene associated with other diseases, namely the Val16Ala and Ile58Thr polymorphisms, are not associated with PD risk [398–400], the Val16Ala polymorphism is linked to PD resulting from exposure to pesticides [398]. In a study using MPTP (1-methyl-4-phenyl-1,2,3, 6-tetrahydropyridine), 3-nitropropionic acid (3-NP), and malonate to induce PD symptoms in mice, Andreassen *et al*. found that heterozygous MnSOD knockout mice have greater dopamine depletion and larger striatal lesions compared to wildtype mice [401]. Liang and Patel found that MPTP treatment results in aconitase inactivation in a time-dependent manner, correlating with an increase in chelatable iron. These effects were assuaged in mice overexpressing MnSOD, providing further evidence that MnSOD expression is an essential factor in PD resulting from environmental toxicants [402].

## 6. Conclusions

**Figure 1:**
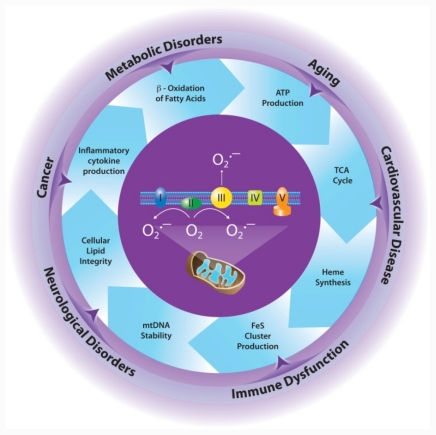
Mitochondria are the major source of ROS in the cell through superoxide production at complexes I and III of the electron transport chain (center). Excessive ROS production can damage different components of mitochondrial metabolic pathways, resulting in altered mitochondrial function and an imbalance in cellular homeostasis (inner ring). Diminished mitochondrial function leads to the development of numerous diseases (outer ring).

Mitochondria play a central role in numerous metabolic pathways in the cell, with functions as diverse as ATP production through oxidative phosphorylation to iron metabolism and utilization. ROS derived from mitochondria can have injurious effects on enzymes involved in these metabolic pathways and other functions of mitochondria, leading to mitochondrial dysfunction and cellular injury/death, culminating in disease development (Figure 1). The mitochondrial antioxidant enzyme MnSOD scavenges superoxide radicals produced within the organelle and can protect mitochondria from the harmful effects of ROS. Methods to increase the expression or activity of MnSOD may prove to be valuable strategies for the treatment of various disease conditions involving aberrant mitochondrial ROS production, including numerous neurological conditions, cardiovascular disease, and cancer that plague countless individuals worldwide.
